# Palliative Care Team Consultation: Circumstances Surrounding Patients Deaths At The Icu

**DOI:** 10.1186/2197-425X-3-S1-A38

**Published:** 2015-10-01

**Authors:** C Biselli-Ferreira, RR Fumis, V Delponte, VR Pizzo, FG Zampieri, DN Forte

**Affiliations:** Hospital Sírio-Libanês, Palliative Care Consultation Team, São Paulo, Brazil; Hospital Sírio-Libanês, Intensive Care Unit, São Paulo, Brazil; Hospital das Clínicas, University of São Paulo, Emergency Medicine Discipline, São Paulo, Brazil

## Introduction

Previous studies reported that patients in end-of-life (EOL) care may still receive aggressive treatment in the Intensive Care Unit (ICU).[1] Despite the fact that less than 10% of Americans report that they would be willing to die in the hospital, up to 45% may ultimately do so. a significant fraction will perish in a critical care setting, which may represent a deviation from previous patients' values. Therefore, it appears to be crucial to understand the circumstances surrounding the death of patients in end-of-life care, specifically which factors might be associated with decision to transfer or to maintain patients in EOL care in the ICU.

## Objectives

To characterize factors associated with death in the ICU in patients accompanied by a palliative care team (PCT) in a tertiary private hospital in Brazil.

## Methods

Retrospective analysis of prospectively collected data based on a standardized questionnaire of PCT. All 628 questionnaires from June 2008 to December 2013 were included. Two groups were compared: patients that eventually died in the ICU versus patients that died outside the ICU.

## Results

Out of 443 (70,5%) patients who died; 76 (12.1%) died in ICU and 367 (58.4%) outside the ICU setting. a comparison between groups is shown in Table 1.

**Table 1:** Table 1

Characteristics	Death at ICU (n = 76)	Death out of ICU (n = 367)	p value
**Age,y, median (SD)**	62.17 (20.34)	66.27 (16.63)	0.102
**Male sex, n (%)**	41 (55.3)	205 (55.9)	0.858
**Cancer diagnosis, n (%)**	53 (69.7)	298 (81.2)	0.037
**Conflicts, n (%)**	27 (35.5)	74 (20.1)	0.006
**Time from referral to PCT until death < 3 days, n (%)**	21 (27.6)	90 (24.5)	0.671
**Time from hospital admission until referral to PCT >14 days, n (%)**	33 (43.4)	119 (32.4)	0.088
**Receiving advanced support of life (mechanical ventilation or vasoactive drugs), n (%)**	32 (42.1)	13 (3.5)	< 0.001
**Patients were in ICU when referral to PCT, n (%)**	53 (69.7)	14 (3.8)	< 0.001
With written directives of end-of-life care *, n (%)	53 (73.6)	311 (87.1)	0.006

**Figure 1:**
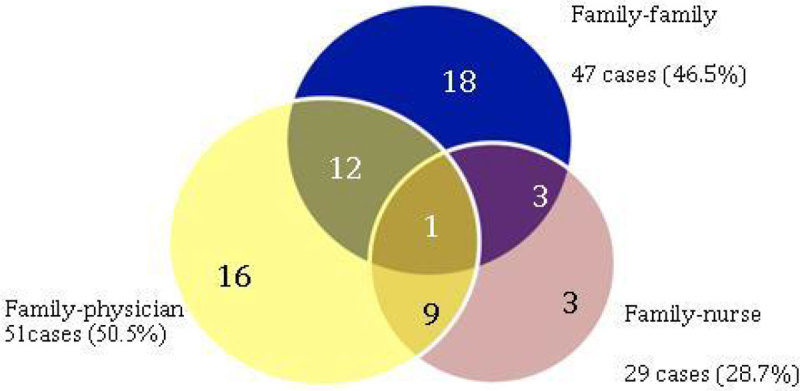
Figure 1

Non-cancer patients died more frequently in the ICU (p = 0.03). We observed the presence of conflicts among patients who died in ICU: conflicts between family members (family-family conflicts) and between family and the attending physician (family-physician conflicts) were equally common, but there were a significant number of conflicts originating between family and nurse staff (Figure 1).

Explicit directives of EOL care were more commonly explicitly written in non-ICU patients (26.4% versus 12.3%, p < 0.006). As expected, we also observed that a remarkable percentage of ICU patients received advanced life support until death (42%). There were no association between death in the ICU and age, late PCT consultation (less than 3 days before death) and prolonged hospitalization (hospital length of stay> 14 days at the of the PCT consultation).

## Conclusions

Circumstances associated with death in the ICU of patients assisted by PCT included a non-cancer diagnosis, conflicts as reason to referral, being in the ICU or receiving life support at the time of the initiation of PCT consultation, and the lack of written advanced directives of EOL care. Further studies are needed to evaluate whether modifying these circumstances may be related to a different EOL care.
